# 55. A rare condition with a persistently unclear aetiology

**DOI:** 10.1093/rap/rky034.018

**Published:** 2018-09-20

**Authors:** Jaspal S Gill, Simon Black, Charles Raine, Pamela Mangat

**Affiliations:** Rheumatology, Royal Free Hospital, London, UNITED KINGDOM

**Introduction:** We present a rare case of macrophage activation syndrome in a previously well 53 year old male with a persistently unclear aetiology. Whilst the patient’s response to anakinra has been consistent with underlying adult-onset Still’s disease, the diagnostic criteria were not clearly met and a concomitant new diagnosis of ankylosing spondylitis raises the possibility of an unusual rheumatological trigger.

**Case description:** A 53 year old male was referred to the infectious diseases team with a three-month history of pyrexia of unknown origin, together with intermittent sweating, rigors and dyspnoea on exertion. The patient had recently been treated successfully for chronic prostatitis with ciprofloxacin although it was felt that this did not entirely explain his symptoms. Past medical history included hypertension but was otherwise unremarkable. Viral and bacterial serology, echocardiogram, plain chest films and lumbar puncture had no abnormal findings. An autoimmune screen was similarly negative and the patient was referred for whole-body FDG PET which showed no abnormal tracer uptake. The patient was subsequently reviewed by rheumatology and, on further questioning, reported stiffness of the back, neck and shoulders. On examination, there was reduced chest expansion, decreased cervical spinal flexion and rotation and Schober’s test was positive at 3cm. MRI of the spine subsequently revealed sacroiliitis and a diagnosis of ankylosing spondylitis was made. On outpatient review, the patient was found to be profoundly diaphoretic, tachypnoeic and tachycardic with a significantly elevated CRP. The patient reported worsening back pain, fevers and a swollen left knee together with an unwitnessed prior rash. The patient was admitted from clinic and, following an increase in oxygen requirements, was subsequently transferred to ITU, where he was intubated. Whilst repeat serology and virology remained negative, plain chest x-ray revealed changes consistent with a rapidly-progressing ARDS and the patient was commenced on empirical antibiotic treatment for presumptive tuberculosis given the initial presentation. Over subsequent days, CRP remained significantly elevated over 300 despite antibiotics and a worsening thrombocytopaenia developed. Bone marrow and trephine biopsy revealed striking haemophagocytosis and the patient required full respiratory support together with cardiac inotropes and haemofiltration. The impression remained of a possible sepsis-driven systemic inflammatory response and multiple antimicrobial therapy continued. However, the patient remained refractory to treatment, including with etoposide, and a primary inflammatory process became more likely as an aetiology. Adult-onset Still’s disease was raised as a differential given the possible history of fleeting arthritis and rash. In this context, the patient’s haemophagocytic lymphohistiocytosis was thought to be macrophage-activation syndrome (MAS) and due to an underlying cytokine storm process. The lack of diagnostic certainty made the decision for immunosuppression difficult and the case was discussed with neurology, intensivists and infectious disease. The consensus was that sepsis of unknown source could not be excluded although, compounding this was the fact that the patient failed to respond to etoposide and high dose intravenous steroids. It was agreed to commence anakinra which was thought to be safe in the context of possible sepsis. The patient became afebrile twelve hours after subcutaneous administration and CRP reduced to 22 over subsequent days. The patient remained in ITU for a 76-day admission with multi-organ dysfunction and slow weaning necessitated an EEG which demonstrated periodic seizure activity. MRI brain confirmed multiple lesions with leptomeningeal enhancement and these were thought to be neurological sequelae of MAS. The patient was commenced on anticonvulsant medication. The patient continued to improve despite multiple pneumothoraces and a left subclavian deep vein thrombosis as a result of intervention. He also suffered from ITU-myopathy which required a period of physical rehabilitation but was discharged from hospital still on anakinra and on a weaning regimen of oral steroids. He has now been switched to methotrexate and remains clinically well.

**Discussion:** This complex case clearly raises numerous interesting diagnostic challenges and uncertainties. The diagnosis of MAS crucially includes the histological finding of HLH on bone marrow biopsy which was present in this case. However, many of the other diagnostic features such as hepatosplenomegaly, hypofibrinogenaemia, multiple line cytopaenia and hyperferritinaemia were not found. MAS is most commonly secondary to juvenile idiopathic arthritis and, whilst evidence suggests it can be found with other rheumatological disease, there are very few cases to support ankylosing spondylitis as a cause. The patient did not convincingly fulfil a diagnosis of adult-onset Still’s given a vague history of rash and persistently normal ferritin, although the presence of arthritis and ongoing pyrexia would obviously not entirely preclude this as an underlying cause, as would the striking clinical response to anakinra treatment. This case raises the rare possibility of ankylosing spondylitis driving a severe systemic inflammatory response but also questions what is known about the interplay between adult-onset Still’s and MAS.

**Key Learning Points:** The need for a multi-disciplinary approach with other specialties, especially infectious disease, when investing and treating complex patient with systemic inflammatory responses to fully exclude sepsis as a driving factor. To consider and explore all possible rheumatological conditions as a trigger for macrophage activation syndrome with the subsequent implications for possible treatment options.

**Disclosure: J.S. Gill:** None. **S. Black:** None. **C. Raine:** None. **P. Mangat:** None.

